# Preschool physics: Using the invisible property of weight in causal reasoning tasks

**DOI:** 10.1371/journal.pone.0192054

**Published:** 2018-03-21

**Authors:** Zhidan Wang, Rebecca A. Williamson, Andrew N. Meltzoff

**Affiliations:** 1 School of Educational Science, Jiangsu Normal University, Xuzhou, Jiangsu, The People's Republic of China; 2 Department of Psychology, Georgia State University, Atlanta, Georgia, United States of America; 3 Institute for Learning & Brain Sciences, University of Washington, Seattle, Washington, United States of America; Purdue University, UNITED STATES

## Abstract

Causal reasoning is an important aspect of scientific thinking. Even young human children can use causal reasoning to explain observations, make predictions, and design actions to bring about specific outcomes in the physical world. Weight is an interesting type of cause because it is an *invisible* property. Here, we tested preschool children with causal problem-solving tasks that assessed their understanding of weight. In an experimental setting, 2- to 5-year-old children completed three different tasks in which they had to use weight to produce physical effects—an object displacement task, a balance-scale task, and a tower-building task. The results showed that the children’s understanding of how to use object weight to produce specific object-to-object causal outcomes improved as a function of age, with 4- and 5-year-olds showing above-chance performance on all three tasks. The younger children’s performance was more variable. The pattern of results provides theoretical insights into which aspects of weight processing are particularly difficult for preschool children and why they find it difficult.

## Introduction

Scientists seek to understand causes, to make predictions, and to change the state of the world. To accomplish this, they construct theories that explain events. A key aspect of theory formation is specifying the causal network underlying events. An accurate and complete account necessarily involves causes that are not obvious and often invisible to the human eye. Preschoolers can reason about causes and effects [1–2], and they have some capacity to consider nonobvious explanations for what they observe [3–5]. In the current experiment, we investigated preschool children’s causal reasoning about the invisible property of object weight and the effects of weight in object-to-object interactions.

Weight (mass) is an invisible property that influences the way in which one object interacts with another object. Weight presents an ideal case for examining how children use an invisible variable to explain observable effects. In contrast to other perceptually salient object properties–such as shape, color, or surface features–weight cannot be seen. Although invisible, weight can influence observable physical effects.

According to classical theories of developmental psychology, young children’s understanding of weight is limited to their kinesthetic experience of objects (i.e., the pressure on their hands and the effort they exert to hold objects) [6, 7]. Piaget postulated that 4-year-olds do not understand weight and have difficulties understanding that two objects that both feel ‘light’ to a human lifter could have different weights. More recent research indicates an earlier understanding of at least some aspects of weight. Even infants can register and use weight differences on problem-solving tasks, as assessed by brain [8] and behavioral [9] measures. For example, after being habituated to an object of a certain weight, 12-month-old infants spend more time interacting with an identical-appearing object of a new weight versus one with the same weight [9]. At about this age, infants also can use visual properties, such as the material composition of objects, to make judgments about the likely weight of an object [10–12]. It has also been shown that 3- and 5-year-olds can predict weight based on an object’s size [13].

Researchers have also examined the development of preschool children’s deeper and more complex understanding of weight. One study showed that 4-year-olds, but not 3-year-olds, differentially categorize visually identical objects according to the underlying weight (they take action to create two piles) after watching an adult intentionally sort objects according to weight [14]. Even more interestingly, a related study found that 4-year-old children in this scenario would actively seek to ‘heft’ the objects themselves, as if to discover an explanation for *why* the adult categorized the identical-looking objects differently, and then the children sorted the objects into underlying ‘kinds’ based on the felt weight [15].

We sought to extend this line of research in causal tasks involving physical interactions between objects. Three types of tasks have been used to examine children’s understanding that the weight of one object can be used to cause effects on another object (‘child physics’). They are: object displacement tasks, balance scale tasks, and tower building tasks [16–18]. The common denominator is that children must consider weight as a property that causally affects how one object interacts with another object.

In displacement tasks, infants as young as 6 months of age display an implicit expectancy that a stationary object will move when it is hit by a moving object [19, 20]. Furthermore, 11-month-olds exhibit looking patterns indicating that they expect a stationary object will move further when hit by a large object versus a smaller object [20]. Preschool children do more than this. They also show an explicit understanding of how weight works in a billiard-ball type collision event. For example, one study showed that children’s decision-making about using an object as a cause is influenced by that object’s weight. In that study children saw an adult launch a heavy and a light object with identical appearances down a ramp, and the prize was dislodged only when hit by the heavy object. When 4-year-olds were given the opportunity to take action to obtain the prize for themselves (i.e., to design a ‘causal intervention’), they choose to use a heavy object to dislodge the prize. 3-year-olds’ performance was not different from chance, suggesting development in children’s understanding of this kind of object displacement task [16].

A second type of task used to explore children’s understanding of weight involves balance scale tasks. Preschool children show some ability to consider what effects different weights may produce when presented with simplified versions of balance scales. For example, when the distance of the weights from the fulcrum is held constant and only the weights are varied, 4- and 5-year-olds, but not 3-year-olds, successfully predict which side of the balance scale will go down [21, 22]. Further, even when the size and appearance of the objects is identical, 4-year-olds choose a heavy versus a light object to tilt down a balance on one side to retrieve a reward under the other side [17].

A third task that is used to study children’s understanding of weight is the tower building task. This assesses children’s grasp of the idea that weight, though invisible, exerts a downward force on a surface, and thus that heavy objects can be supported by certain surfaces but not others. By the preschool years, children have developed expectations about what materials can support different weights. For example, 3-year-olds believe that a heavy object, but not a light object, will make a foam rubber bridge collapse [18], and they are more likely to make a decision to choose a metal versus a paper platform to support a heavy object [16]. Thus, there is an understanding that the composition of materials and weight interact in certain predictable ways.

Developmental scientists’ understanding of how young children think about weight as an invisible causal property is still relatively limited. It is clear that the preschool age period is important in the development of children’s understanding of weight [16, 18, 23]. However, there are two interrelated questions regarding weight as a causal variable that are largely unaddressed in the extant literature. First, the age at which children can *consistently and generatively* intervene to use weight as a causal variable to produce effects (in the absence of linguistic or visual clues) is not clear. Previous work suggests that the preschool period is important, but multiple tasks have not been systematically studied in the same children using well-controlled experimental studies.

Second, prior findings document heterogeneity in children’s use of weight to produce effects and hint that children’s understanding of weight as a cause may be ‘graded’ rather than ‘all or none.’ Similar findings were found in the related realms of children’s understanding of solidity [24–26]. In one study, a ball rolled down a ramp, disappeared behind an opaque screen, and was stopped by a vertical barrier. The vertical barrier could be positioned at one of several places behind the screen. The children were then asked to find the ball. Children had to use the top of vertical barrier, which extended several centimeters higher than the screen, to determine where the ball would be stopped and thus to make the correct judgments of where to find it. The results showed that 3-year-olds, but not 2.5-year-olds, were able to solve this problem in a consistent manner [24]. However, when either: (i) the opaque screen was replaced by a transparent one [25] or (ii) the children were allowed complete visual access to the movements of the ball before the opaque screen was lowered to conceal it [26], 2.5-year-olds’ performance was significantly increased. Therefore, and in a similar fashion, another goal of the current study is to systematically investigate the degree to which children’s understanding of causal reasoning tasks involving weight may differ as a function of theoretically-driven variations in the nature of the task.

The three standard weight tasks described earlier (displacement, balance-scale, and tower-building) have not been tested in the same children. Here we used a within-subjects design to test whether children begin to use weight to produce effects in all three tasks at the same age. Although weight is the crucial factor in all three, the way in which weight exerts the effects in each task is different. In the object displacement task, the single event is a displacement which is the result of a direct spatiotemporal interaction (collision) between two objects. In the balance scale task, the influence of the heavy and light object are at a distance (at the end of the balance arm) and there are multiple events—the balance scale tips and the target toy is revealed as one of the sides lifts up. Moreover, in both of these weight tasks, there is a salient perceptual transformation in the environment (e.g., the prize is dislodged from the ramp, the balance scale is tipped). In contrast, to succeed in the tower building task, the child must use inhibitory control to avoid the visually salient event (the heavy object crashing through the sponge platform) and select the light object to achieve the final goal of building a tower.

Previous studies point to preschool as an important developmental period in children’s understanding of weight, and therefore, we systematically tested children at 2, 3, 4, and 5 years of age. We expected significant age-related development in understanding weight as a function of task across this period. Based on the extant literature, we expected that the 2-year-olds may be able to solve the object displacement task (predicted to be the easiest of the three), but that children would not be able to generatively solve all three tasks until approximately 4 years of age. In the Discussion we provide ideas about why this may be the case and the possible role of language in helping children come to a deeper and more generative understanding the invisible property of weight.

## Methods

### Participants

The sample consisted of 72 children. There were 18 children at each of four different ages: 2 years of age (*n* = 18; 9 males), 3 years of age (*n* = 18; 8 males), 4 years of age (*n* = 18; 12 males), and 5 years of age (*n* = 18; 8 males). According to parental report, the sample was 83% white, 7% Asian, 3% Black/African American, 3% other, and 2% not reporting, with 2% as being of Hispanic ethnicity. The study was approved by Georgia State’s Institutional Review Board (IRB). In accordance with the institution policy for testing of human children in a museum setting, oral consent was obtained from all parents prior to testing. The general age range of the children was recorded based on the parent(s)’ report, because the Georgia State’s IRB did not allow us to confirm the children's exact date birth.

### Apparatus

**Fig 1:**
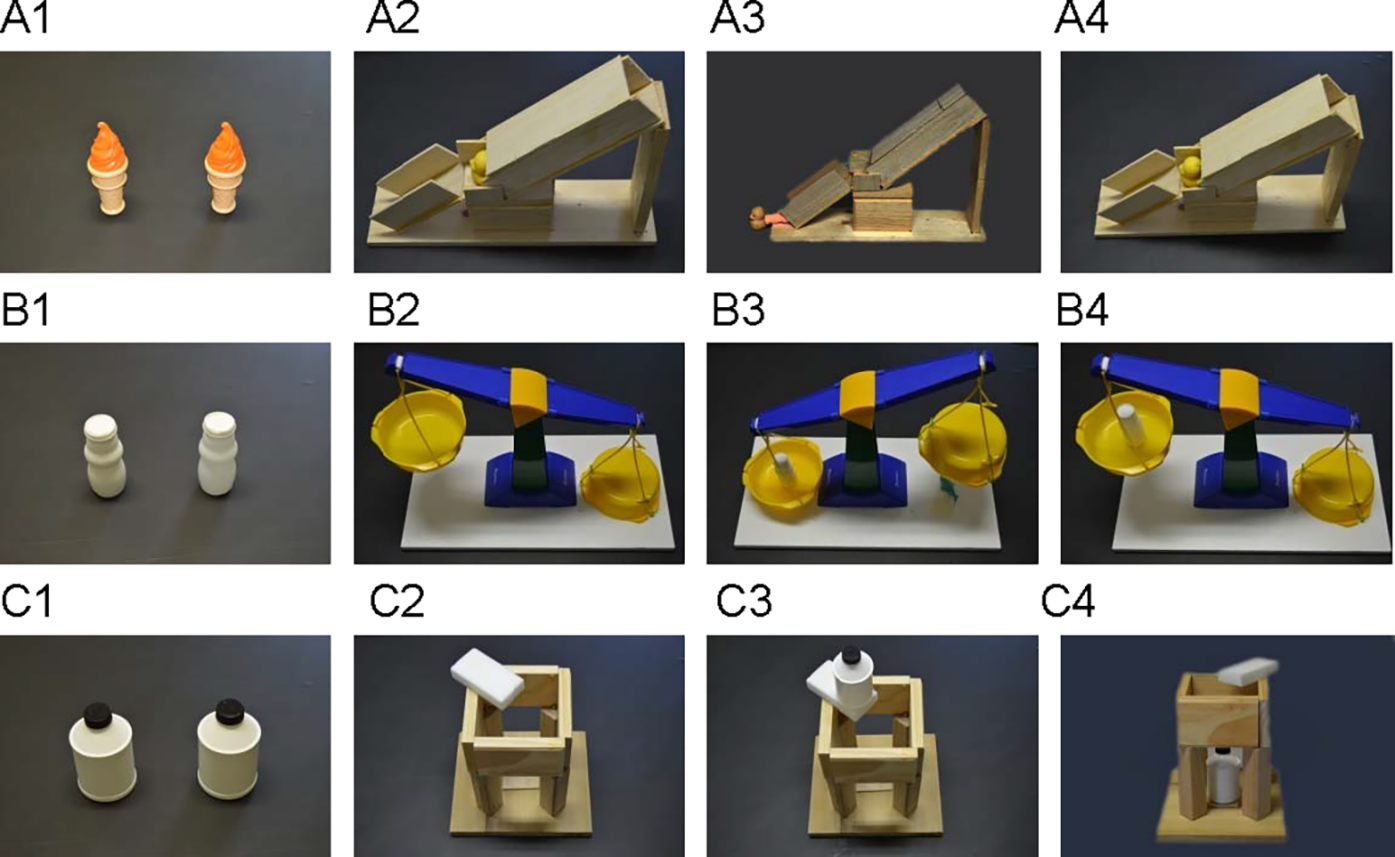
Three tasks used in the experiment. The object displacement task is shown in row A; the balance scale task in row B; and the tower building task in row C. In each row, the picture in column 4 shows the outcome when the non-target object is used (the light one in A4 and B4; and the heavy one in C4).*Note*: The objects in picture A3 at the bottom of the chute are the duck and the base of the cone (the child inserted the cone so that the light orange base of the cone was oriented to slide down the chute first, thus dislodging the duck).

Two objects have an ice cream cones shape (1 ×1 ×5 inches) were used as the objects for launching in the object displacement task. The heavy ice cream cone weighed 94.41g, whereas the light ice cream cone weighed 15.35g. Two jugs (1.25 ×1.25 ×4 inches) were used for the balance scale task. The heavy jug weighed 147.20g and light one weighed 6.24g. Two capped bottles (3 ×3 ×5 inches) served as the objects that would be supported for the tower building task. The heavy bottle weighed 588.80g, whereas the light bottle weighed 25.64g (Fig 1).

#### Object displacement task

The object displacement chute apparatus (22 ×6×15 inches) was made of a wooden ramp with a cover of wooden planks. A yellow duck was placed on a small platform in the middle of the ramp so that it would be displaced when the heavy object collided with it. The yellow duck was visible on the platform, however, the actual collision was invisible because the covering of the chute masked it from view. The weighted objects were launched through a hole at the top of the ramp. The mass of the heavy object was such that it dislodged the yellow duck from the middle platform, and both arrived at the bottom of the apparatus. The light object did not dislodge the duck and both objects remained trapped in the middle platform (Fig 1A).

#### Balance scale task

A two-armed balance scale (24 ×12 ×15 inches) was located in the middle of a white board. On the left arm of the balance was a yellow bowl, which was used to cover the target toy from the participant’s view. On the right arm of the balance was another yellow bowl, in which specifically weighed objects could be placed. The yellow bowls on the arms of the balance scale looked similar, however, the bowl on the right was heavier than the bowl on the left, so that the scale tilted down to the right in the initial state. When the heavier of the two test objects (the white jugs) was placed on the left bowl, it tilted the balance scale’s arm down to the left, revealing the target toy under the right bowl. The light object did not successfully tilt down the balance scale and the target toy remained hidden under the right bowl (Fig 1B).

#### Tower building task

Four vertical wooden pillars were attached to a flat wooden board. Four other wooden pieced were mounted to form a square on the top of the pillars. The overall apparatus measured 12 ×10 ×11 inches. The light object could be supported by the white soft sponge, but the heavy object could not be supported by the sponge material and dropped into the inside of the apparatus (Fig 1C).

## Procedure

The general procedure was similar for each task. We used an ‘observation-only’ design [27, 28] in which the participants did not have any first-hand manipulative experience with the objects before they were administered the critical test in the response period. They simply observed the adult’s actions during the demonstration period [28] and were first allowed to touch and handle the objects during the response period.

The experimenter drew the children’s attention to the apparatus. To illustrate how the apparatus worked, the experimenter showed the children how to use the objects in each of the problem-solving tasks and showed the effects produced by each of the two objects. Unbeknownst to the child during the demonstration (or not known through first-hand manipulative experience), one of the objects was heavy and the other was light. Verbal labels about weight/size/material were not used. The children observed the object-object interaction and were not allowed to touch the objects or the apparatus as they watched the demonstration. Then, the child was allowed to design their own ‘causal intervention,’ that is, to choose to use one of the objects in order to bring about the effect themselves.

Each child had a total of four trials for each task. Because there were three tasks, each child received 12 trials in total. The following two factors were counterbalanced across participants: (i) the order in which the heavy versus light object was used first in the demonstration, and (ii) the order of the tasks. We also counterbalanced across trials whether in the response period, the heavy or light object was placed in the child’s right versus left hand. Thus, any differences in performance were attributable to the task rather than these factors. This study used a within-subjects design for the three experimental tasks with similar presentation and response periods for each. Each is described in detail below.

### Object displacement task

#### Demonstration phase

The goal of this task was to test whether children understand that when one object collides with another, the object displacement caused by the moving object varies as a function of the moving object’s weight (the stationary object is controlled). The experimenter drew the children’s attention and pointed at the duck, and said, ‘Look, there is a duck. I am going to get the duck.’ After the children noticed the location of the duck, the experimenter picked up the two weighted objects (cones) and launched them one at a time from the top of the ramp. When the heavy cone was launched, the duck was displaced from the middle platform and arrived at the bottom of the ramp. After the duck reached the bottom, the experimenter picked it up and said, ‘Look, I got the duck.’ However, when the light cone was launched from the top of the ramp, the duck and the cone both were trapped at the middle platform. The experimenter said, ‘Look, I did not get the duck’.

#### Response phase

The experimenter set up the chute, gave the two objects to the child, and said, ‘Now it is your turn to get the duck.’ The child was asked to choose one object to use with the apparatus. If the child chose to use the heavy object to launch down the ramp, both the object and the duck arrived at the bottom of the ramp. The experimenter told the children, ‘You got the duck. You can play one more time.’ The child was allowed to keep the duck in front of them. The purpose of allowing the child keep the duck was to encourage them to try to cause the effect and acquire the duck. (In either case, whether the participant used the heavy or light object, between each trial, the experimenter moved the cones away from the chute, and reset the apparatus by placing a new duck at the middle platform before giving the child another two objects to use, and repeated this for a total of four trials.) If the child launched the light object, both the weighted object and the duck were trapped in the middle platform. The experimenter told the children, ‘You did not get the duck. You can play one more time’.

Importantly, the procedure was designed so that the child could not *visually track* which was the heavy and which was the light object after the adult’s demonstration or between trials. After the demonstration and between trials, the experimenter moved the two objects behind him (the experimenter and the child sat face-to-face, so when the experimenter put objects behind his body, the child’s visual contact with the objects was broken). When the adult brought the objects out from behind his back, the position of the heavy and light object was counterbalanced such that for half of the trials the heavy object was placed in the child’s right hand and the light object was placed in the child’s left hand; and for half of the trials it was the reverse. Thus the child could experience and compare which was the heavy versus light object (each hand held one weight), and the child had a goal to achieve (to obtain the duck). The child’s problem was to choose which of the two visually identical objects to use to achieve the goal–the heavy or light object–and to perform the correct intervention to bring about a change in the physical world. This same logic, and same break in visual contact, so that visually tracking the heavy and light objects was impossible, was used for the balance scale and tower building tasks described below.

### Balance scale task

#### Demonstration phase

In this task, we examined whether children understand that weight can affect the arm of the balance scale. The experimenter first drew the children’s attention to the apparatus. He tilted down the balance scale manually by pushing the right arm of the scale to reveal the hidden toy and said, ‘Look, there is a little shark. I am going to get the shark.’ After that, he picked up the two weighted objects (jugs) and placed them one at a time in the bowl of the balance scale. When the heavy object was placed into the left bowl, the balance was tilted down on the left side, revealing the prize (a toy shark) under the bowl on the right side. The experimenter picked up the shark and said, ‘Look, I got the little shark.’ When the light object was placed in the bowl, the balance maintained its original position. The experimenter said, ‘Look, I did not get the little shark’.

#### Response phase

The experimenter set up the apparatus, gave the two objects to the children, and said, ‘Now it is your turn to get the shark.’ They were prompted to choose one object to put into the balance scale. The verbal instructions were similar to the previous task, and four trials were administered.

### Tower building task

#### Demonstration phase

The goal of this task was to examine children’s understanding of how weight impacts a support made of a certain material. The experimenter introduced the apparatus by specifying the goal of the task and saying, ‘Look, I am going to build a tower.’ After that, the experimenter picked up the two weighted objects (plastic bottles) and placed them one at a time on the sponge platform. When the light one was placed on the platform, it sat stationary on the sponge because the platform could support it. When the heavy one was placed on the support, it fell into the apparatus because the platform could not support it. To build a tower on the sponge platform, children had to choose to stack *light* object instead of a heavy one. The verbalizations were again similar to the previous tasks.

#### Response phase

The experimenter set up the apparatus, gave the two objects to the children, and said, ‘Now it is your turn to build the tower.’ Children were prompted to place one of the objects on the sponge platform. If the child placed the heavy object on the platform, it would fall into the cube. The experimenter said, ‘You did not build the tower. You can play one more time.’ As in the previous tasks, four trials were administered.

### Dependent measure and scoring

For the object displacement and the balance scale tasks, the dependent measure was the number of trials in which the child chose the heavy object. If the child chose the heavy object he/she was scored as a 1 on that trial, yielding a score ranging from 0–4 for each task (1 possible point for each of four test trials). For the tower building task, the dependent measure was the number of trials in which the child chose the light object. If the child chose the light object he/she was scored as a 1, yielding a score ranging from 0–4 (see S1 File). We used the light object as the correct answer in this task to provide variety, so that the correct answer was not always to pick the heavier of the two objects.

The primary scorer recorded all the children’s responses online, recording a yes/no judgment as to whether the children first placed the correct object into the apparatus. Specifically, for each trial, the scorer indicated whether the effect was produced (e.g., the duck was dislodged, or the toy was revealed beneath the scale arm), and which hand the chosen object was placed in. A research assistant, who not involved in this research and unaware of the theory or hypotheses, also recorded the responses of 20% of randomly-selected participants. Interrater agreement on the child’s object choice was high as assessed using Cohen’s kappa (*k* = .92).

## Results

Preliminary analyses showed no significant effects of the child’s gender, task order (the object displacement task, the balance scale task, or the tower building task first), or the order in which each object (heavy vs. light) was used first in the demonstration. Thus these three factors were collapsed in all subsequent analyses. Across tasks, the correct object chosen scores met assumptions of normality (Normality test using skewness, *z* = 2.46, *p* > .05, and kurtosis, *z* = .10, *p* > .05), but violated Mauchly's test of sphericity; χ^2^(2) = 24.07, *p* < .001. Therefore, Greenhouse-Geisser adjusted degrees of freedom are reported.

We first conducted a 4 (Age: 2-, 3-, 4-, 5-year-olds) × 3 (Task: object displacement, balance scale, tower building) analysis of variance (ANOVA). The results indicated that there was a significant main effect of age, *F*(3, 68) = 13.42, *p*< .001, *η*_*p*_^*2*^ = .37 (2-year-olds: *M* = 2.54, *SD* = 1.28; 3-year-olds: *M* = 2.83, *SD* = 1.31; 4-year-olds: *M* = 3.44, *SD* = 0.98; 5-year-olds: *M* = 3.72, *SD* = 0.62). Follow-up Student-Newman-Keuls comparisons showed that 5-year-olds chose the correct weight to produce the desired effect significantly more than either 2-year-olds (*p* < .001) or 3-year-olds (*p* < .001). Also, 4-year-olds did this significantly more than either 2-year-olds (*p* < .001) or 3-year-olds (*p* < .001). There was no significant difference between 2- and 3-year-olds (*p* = .16), nor was there a difference between 4- and 5-year-olds (*p* = .19). There was no significant main effect of task, *F*(1.54, 104.47) = 1.52, *p* = .23 and no significant age × task interaction, *F*(4.61, 104.47) = 1.14, *p* = .34.

For completeness, we also adopted a nonparametric statistical approach and employed Kruskal-Wallis tests for comparisons, and found similar results. The results showed a significant difference of age in total correct object choice scores, *χ*^*2*^(3) = 28.67, *p* < .001. Subsequent Mann-Whitney *U* pairwise comparisons revealed that 5-year-olds chose the correct weight to produce the desired effect significantly more than either 2-year-olds (*p* < .001) or 3-year-olds (*p* < .001). Also, 4-year-olds did this significantly more than either 2-year-olds (*p* < .001) or 3-year-olds (*p* = .012). There was no significant difference between 2- and 3-year-olds (*p* = .09), nor was there a difference between 4- and 5-year-olds (*p* = .14).

The above analyses suggest that the way children use weight to bring about a change in the physical world develops with age. To determine the age at which children can solve all the three tasks, children’s performance was compared to chance. In each trial, two options were presented. Thus, if the child randomly chooses one object to produce the effect, (s)he has a 50% chance of choosing the correct object. Considering that there are four trials, chance performance is a target score of 2 (4 trials × .50).

**Fig 2:**
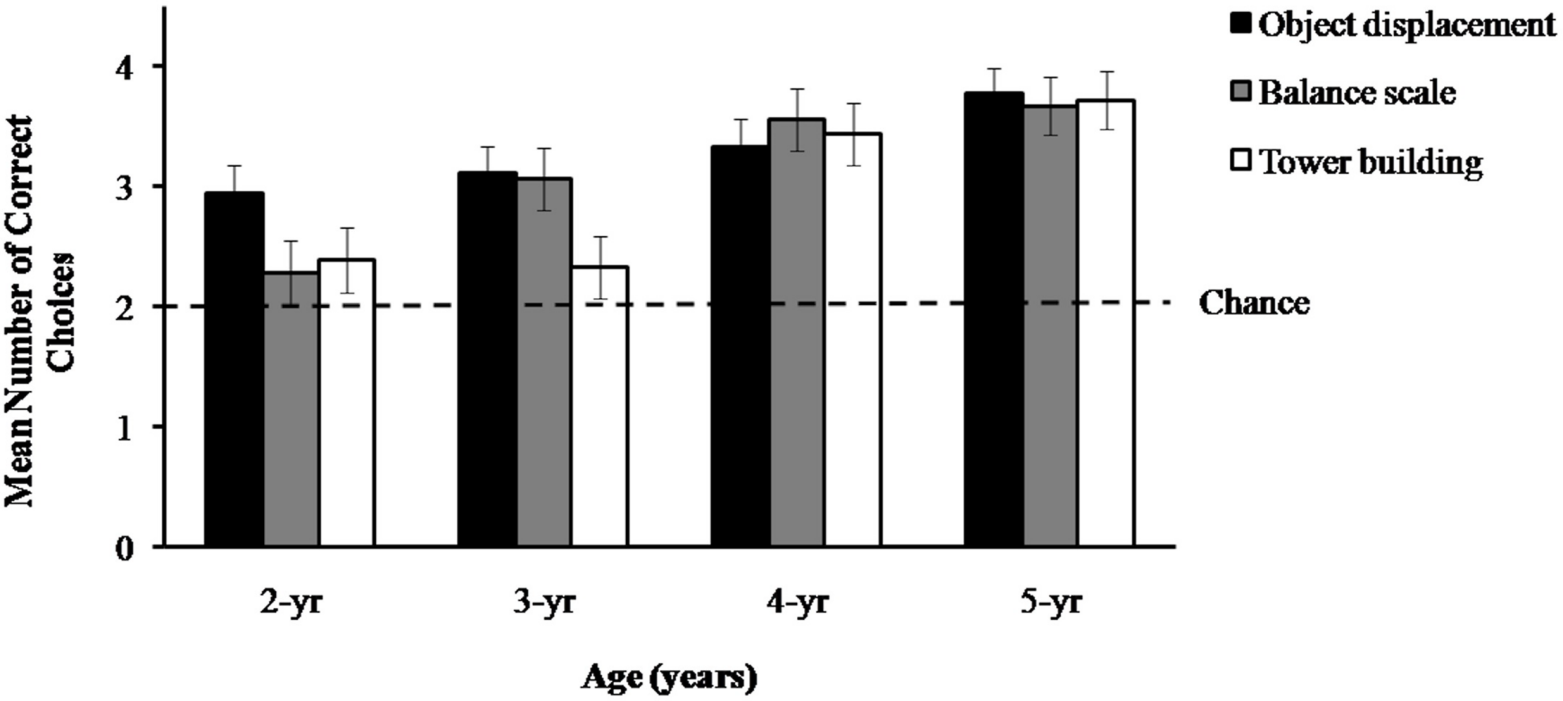
Mean correct choices (+/−SE) as a function of age and task.

As shown in Fig 2, 2-year-olds as a group performed significantly above the chance value of 2 on the object displacement task, *t*(17) = 3.61, *p* = .002, *d* = .85, but not on the balance scale, *t*(17) = .89, *p* = .38, or tower building task, *t*(17) = 1.16, *p* = .26. The 3-year-olds performed significantly above chance on both the object displacement, *t*(17) = 3.93, *p* = .001, *d* = .90, and the balance scale task, *t*(17) = 3.70, *p* = .002, *d* = .88, but not on the tower building task, *t*(17) = .95, *p* = .36. Both 4- and 5-year-olds performed significantly above chance on all the three tasks (4-year-olds: object displacement task, *t*(17) = 5.22, *p* < .001, *d* = 1.22; balance scale task, *t*(17) = 6.71, *p* < .001, *d* = 1.59; tower building task, *t*(17) = 7.16, *p* < .001, *d* = 1.67. 5-year-olds: object displacement task, *t*(17) = 17.63, *p* < .001, *d* = 4.14; balance scale task, *t*(17) = 7.29, *p* < .001, *d* = 1.72; tower building task, *t*(17) = 15.85, *p* < .001, *d* = 3.74).

A Friedman test was conducted to assess whether children used weight similarly across the four test trials (1–4) (Table 1). Collapsing across the three tasks, children’s use of weight improved significantly over the four trials in the tests, *χ*^*2*^(3) = 19.99, *p <* .001 (the values for the four trials were, respectively: Trial 1: *M* = 2.14, *SD* = 0.79; Trial 2: *M* = 2.35, *SD* = 0.79; Trial 3: *M* = 2.40, *SD* = 0.71; Trial 4: *M* = 2.51, *SD* = 0.71). For completeness, we also broke these same data down to the level of the individual tasks. Tests for each task considered separately showed that children’s use of weight did not significantly vary over the four trials for the object displacement, *χ*^*2*^(3) = 5.11, *p =* .16, or the balance scale task, *χ*^*2*^(3) = 5.16, *p =* .16; however, children’s performance showed significant improvement across trials for the tower building task, *χ*^*2*^(3) = 17.19, *p =* .001.

**Table 1:** Mean correct choices (+/− *SD*) as a function of task and trial.

	Trial 1		Trial 2		Trial 3		Trial 4	
	*M*	*SD*	*M*	*SD*	*M*	*SD*	*M*	*SD*
Object displacement task	0.75	0.44	0.85	0.36	0.83	0.38	0.86	0.35
Balance scale task	0.75	0.44	0.82	0.39	0.74	0.44	0.83	0.38
Tower building task	0.64	0.48	0.68	0.47	0.83	0.38	0.82	0.39

## Discussion

In this experiment, children’s knowledge of intuitive physics was investigated using three causal reasoning tasks. In each task, children needed to solve a problem that involved object-object causal relations involving weight. The children needed to determine which of two differently weighted objects should serve as a ‘cause’ to produce an effect on another object and then to act (design an ‘intervention’) to bring about the desired outcome.

Three key results emerged. First, children’s use of weight to produce effects improved with age: 4- and 5-year-olds chose the objects to produce the desired effects significantly more often than either the 2- or 3-year-olds. Second, by the age of 4 years children consistently solved all three tasks. 3-year-olds performed above chance level only on the object displacement and the balance scale tasks, and 2-year-olds performed above chance level only on the object displacement task. Third, for the tower building task (which was the most difficult one), children’s use of weight significantly improved across the test trials.

The novelty of this study includes: (i) the within-subjects design comparing three tasks to one another, (ii) exclusion of linguistic cues referring to weight, (iii) strict experimental procedures in which visually tracking of the efficacious object (heavy vs. light) was ruled out, and (iv) systematic examination of age effects by using the same procedures and tasks across four ages. Most of the previous research investigated only a single task, and different studies used variations in how tasks have been presented. The methodological features used here contribute to our understanding of how children’s use of weight as a cause develops as a function of age.

Crucially, children were solving these causal problems and taking action to bring about changes in the physical world by harnessing the invisible property of weight. During the demonstration the children saw the adult succeed on the task using one of two objects, and saw the adult fail on the task using another object. However, the adult refrained from using any linguistic descriptions about weight/amount/size, and the children were blocked (by procedural design) from visually tracking which individual object of the two visually identical ones was the one that had been efficacious. The child simply watched the event and that ‘set’ the problem for the child to solve. The child then was given the objects and the child themselves spontaneously chose the ‘correct answer’ (heavy vs. light object) to achieve the goal that had been set *visually* by the adult.

One aim of the current experiment is to determine the age at which children consistently use weight to produce effects on each task. The results suggested that 4- and 5-year-olds chose the correctly weighted object to produce the desired effects significantly more often than either the 2- or 3-year-olds, and there was no significant difference between 4- and 5-year-olds. Comparing children’s performance with chance showed that 4-year-olds solved all three tasks, whereas 3-year-olds did not solve the tower building task and 2-year-olds did not solve either the tower building or balance scale tasks. Thus, by age 4, children can reliably understand weight as a cause across a variety of different causal reasoning tasks.

We found that surprisingly young children (2-year-olds) had the ability to use weight as a causal variable to bring about a change in the physical world, even when no relevant language was used and the choice was between two visually *identical* objects—though they only did so under restricted conditions. More specifically, even the 2-year-olds solved the object displacement task, but they failed the other two tasks.

Previous studies have shown that young children succeed on causal reasoning tasks, but these have tested causal reasoning outside of a weight context [29–32]. In one paradigm, children are introduced to a machine called a ‘blicket detector.’ They are shown that the machine lights up and plays music when some objects (‘blickets’), but not others, are placed on it. Children are thus confronted with a novel causal relation. When told that one of the objects that can make the machine light up and play music is a blicket, children as young as age 2 to 3 years old extend the verbal label ‘blicket’ to objects that produced the same effect on the machine [29]. Other causal reasoning tasks have also shown that 2-year-old children can determine which of two similar objects are causally efficacious even when causal language and spatial contact information is ruled out [28, 32]. But again, the crucial variable tested in this work did not involve weight. In the current experiment, we show that children as young as two years of age use weight to design interventions and bring about effects in the physical world, much earlier than predicted by Piaget [6].

However, our results also suggest that the 2-year-old children’s capacity to reason generatively about causal relations related to weight does not match the adult conception. The age period from 3 to 4 years old is an important transition period for using weight more generally across a wider range of tasks. These results are consistent with other studies outside the area of weight understanding, which documents differences between younger and older preschool children’s depth of reasoning about causal relations across different contexts [33, 34]. What, then, accounts for the developmental difference?

It is not likely, for example, that the younger children failed to solve the difficult task because they lacked any knowledge about supporting/collapsing. One-year-olds show some implicit understanding that heavy objects compress cotton wool more than light object [11]. It remains possible, however, that the younger children cannot use their physical knowledge to select, plan, or guide appropriate action in the causal reasoning task [17]. For example, in order to plan and execute a differentially successful action in the tower task, some degree of inhibitory control is needed to use the light object and skip over the heavy one (especially since they both have the same appearance). In addition, it is possible that between ages 3 and 4 years, children come to understand in a new more general way that an *internal/invisible property* such as weight has causal powers that underlie and explain visible outcomes, which would generally comport with other studies [35, 36]. For example, in one study, children saw that an object with one internal part would make a machine light up, whereas the same object with a different internal part did not produce this effect. When asked to predict which of two novel objects would make the machine light up, it was not until age 4 that children consistently understood that a specific internal/invisible property was necessary to achieve the desired effect [36].

A third account of the age-related change is that the development of language contributes to the understanding of weight as an invisible cause. Prior child developmental research shows that older children are more likely to generate verbal explanations and to share these explanations with the adult [17]. There are at least two ways in which talking about weight during everyday interactions (before the experiment) may help children learn about the invisible property of weight as a cause. First, discussing weight cause-and-effect, and particularly attempting to explain events involving weight as a cause, may draw children’s attention focus to key aspects of the task. Second, in Brown’s felicitous words [37], language may act as ‘an invitation to form concepts,’ and the abstract symbol system of language may be especially suited to promoting cognitive growth about *invisible* causes such as weight [38, 39].

We also found success varied across the different task contexts and so the understanding of weight was not an all-or-none conceptual acquisition (this result is consistent with the finding of a graded developmental understanding in the realm of children’s reasoning about solidity and continuity) [24–26]. This necessarily leads to a consideration about why some of the weight tasks are developmentally more difficult than the others. The balance scale task was more difficult for children than the object displacement task; we also found that the tower building task was more difficult than both of those. Why?

In the object displacement task, the single event is the displacement caused by a collision (albeit an invisible one, see ‘Method‘). In the balance scale task, however, there are multiple events—the balance tips and the target toy is revealed. It has been shown that the number of events in a single decision influences children’s reasoning about variables and their interrelations. It might be argued that the cognitive load is heavier [40, 41] in the balance scale task because it involves many pieces of information and moreover the physical interaction involves the relative balance of two objects at a distance from one another and not a direct collision. Further studies should systematically manipulate the number of events in each task to examine how these factors influence children’s performance.

Finally, we wish to offer thoughts about the difficulty of the tower building task. In both the object displacement and the balance scale tasks, the desired goal is achieved when the heavy object is used. When the heavy object is placed on the ramp it dislodges the duck, and when heavy object is placed on the balance scale, it tips. However, achieving the desired effect in our tower building task entailed placing the light object on the sponge platform. There is evidence that boosting the perceptual salience of variables can make children more likely to consider causal relations among the variables [33, 42]. Children’s conception of the world may be such that solving a weight task is easier when there is a perceptually salient outcome tied to weight. Young children may think that *heavier weight = more work = more visible change* in the environment. On this conception, the tower building task may be more difficult for children to understand (cognition) or achieve (motivation), because the desired effect of building the tower is less salient than the perceptual effects of the collision or tipping of the balance to reveal a prize.

Children’s performance in the tower building task (but not the other tasks) increased over trials, and this could be because they had to *avoid* using the heavy object which had worked to produce the outcome in the other tasks; however, contra this interpretation, we did not find a significant task-order effect. Therefore, we tentatively offer the speculation that this tower building task may be difficult because the children needed to conceive of the *same* support structure (the sponge) in two *contradictory* ways, both as supporting the object and not supporting it, depending upon the object weight. Being able to think that the same object (the sponge) in two ways—that is, it both can and cannot support visually identical objects depending on the weight of the to-be-supported object—may be taxing for young children. At the same time, we acknowledge that the ‘effect’ (the object stays stationary on its support to build a tower) may not have been as enticing as the effect in the other two tasks, because it did not involve as salient a change in the visual environment.

## Conclusions and wider theoretical implications

The current research addressed whether and when children can apply their understanding of the effects of the invisible property of weight across three divergent tasks. The results show that by age 4 children use weight as a causal variable on all three tasks. The results further suggest interesting changes as a function of age, inasmuch as the object displacement task was solved at the youngest age, 2-year-olds. Future research will need to be directed at two chief developmental issues raised here: (i) how children build on their nascent understanding of object-object relations in the object collisions (heavy objects can displace other lighter objects) as a foundation for developing an understanding of the more difficult tasks involving weight (balance scale and tower building), and (ii) the role that language may play in children’s developing understanding of how weight works in a generative fashion to cause a range of disparate effects in the visible world. Although language concerning weight was not used during the experiment (words concerning mass, amount, size were excluded), it is likely that children’s understanding of invisible forces such as weight develops and expands through their mastery of the symbol system of language and by participating in everyday discussions endeavoring to explain the object-object interactions that they see in the world.

A final intriguing theoretical issue raised by the current findings concerns the developmental shift that occurs at about 4 years of age and its possible relation to the emergence of explicit theory of mind reasoning at about this same age [43]. The weight tasks, like the explicit theory of mind tasks, requires more than changes in visual attention as measured by looking-time scores. In the explicit theory of mind literature, children need to describe and justify their choices; in the current experiment children need to make a choice between two objects to take action (perform an ‘intervention’) designed to bring about a change in the physical world based on the invisible property of weight. In both cases, children need to make decisions based on invisible properties (weight in relation to things; and mental states in relation to intentional agents). It would be interesting to directly compare children’s understanding of the invisible property of weight and invisible mental states in the same children in the same test session. Based on the advances in this paper and others, we now have tools to conduct longitudinal studies comparing children’s intuitive theories of both physical causality and social causality—a topic of central importance in developmental science [16, 23, 32, 44].

## Supporting information

S1 File DataFile. Data set that includes the children’s correct choice scores of the object displacement, balance scale, and tower building tasks.
(SAV)
